# A planning study for palliative spine treatment using StatRT and megavoltage CT simulation†

**DOI:** 10.1120/jacmp.v12i1.3348

**Published:** 2010-10-30

**Authors:** Yi Rong, Poonam Yadav, Bhudatt Paliwal, Lu Shang, James S. Welsh

**Affiliations:** ^1^ Department of Human Oncology University of Wisconsin ‐ Madison Madison WI USA; ^2^ Department of Medical Physics University of Wisconsin ‐ Madison Madison WI USA; ^3^ University of Wisconsin Riverview Cancer Centre Wisconsin Rapids WI; ^4^ Vellore Institute of Technology University Vellore Tamil Nadu India; ^5^ Guangxi Polytechnic of Construction and Technology Guangxi China

**Keywords:** helical tomotherapy, StatRT planning, megavoltage CT imaging, palliative treatment

## Abstract

Megavoltage CT (MVCT) simulation on the TomoTherapy Hi·Art system is an alternative to conventional CT for treatment planning in the presence of severe metal artifact. StatRT is a new feature that was implemented on the TomoTherapy operator station for performing online MVCT scanning, treatment planning and treatment delivery in one session. The clinical feasibility of using the StatRT technique and MVCT simulation to palliative treatment for a patient with substantial spinal metallic hardware is described. A patient with metastatic non‐small‐cell lung cancer involving the thoracic spine underwent conventional kilovoltage CT simulation. The metal artifact due to stainless steel spine‐stabilizing rods was too severe for treatment planning, despite attempts to correct using density override. The patient was then re‐scanned using MVCT on a tomotherapy unit. Plans were generated using both StatRT and conventional tomotherapy planning (Tomo plan) with different settings for comparison. StatRT planning ran a total of five iterations in a short planning window (10–15 min). Two Tomo plans were generated using: (1) five iterations in the “full scatter” mode, and (2) 300 iterations in the “beamlet” mode. It was noted that the DVH of the StatRT plan was almost identical to the Tomo plan optimized by the “full scatter” mode and the same number of iterations. Dose distribution analysis reveals that these three planning methods yielded comparable doses to heart, lungs and targets. This work also demonstrated that undermodulation can result in a high degree of thread effects. The overall time for the treatment process (including 7 minutes for simulation, 15 minutes for contouring, 10 minutes for planning and 5 minutes for delivery) decreases from hours to around 40 minutes using the StatRT procedure. StatRT is a feasible treatment‐planning tool for physicians to scan, contour and treat patients within one hour. This can be particularly beneficial in urgent palliative treatments.

Conflict of Interest Statement: James S. Welsh has received honoraria for speaking for TomoTherapy, Inc.; Yi Rong has received travel sponsorship for TomoTherapy, Inc.

PACS numbers: 87.55.D‐, 87.57.C‐

## I. INTRODUCTION

The use of computed tomography (CT) in radiation therapy has been largely increased due to the essential role of the accurate 3D anatomical information and the attenuation coefficient data that allow tissue heterogeneity corrections in the treatment planning process. Though the vastly improved kilovoltage CT (kVCT) scanners have 3D imaging capabilities and have reduced the scanning time, metallic artifacts persist in the present generation of CT. CT artifacts have many causes including patient movement, the limitation of the detector, the image reconstruction method, and the presence of high‐density materials such as metal in or on the patient.^(^ ^1^ ^)^ Streak artifact is one of the most significant challenges in the application of diagnostic kVCT simulation. It can be caused by one or more effects such as beam hardening, scattered radiation, poor signal‐to‐noise ratio, aliasing and object motion, which may result in significant bright and dark streaks on the reconstructed image.^(^ ^2^ ^)^ Metals can also cause streak artifacts, the main cause of which is the high attenuation and scattering properties of metal in diagnostic X‐ray energy range. A direct solution is to use less‐attenuating materials (e.g., titanium) or devices with smaller scatter cross‐section.^(^ ^3^ ^–^ ^5^ ^)^ However such case‐by‐case solutions are not ideal for clinical practice. Promising mathematical algorithms were also proposed for artifact reduction on diagnostic CT images.^(^ ^6^ ^,^ ^7^ ^)^ But manufacturers have not yet implemented them for clinical use. In radiotherapy application, metal artifacts greatly limit the usefulness of kVCT images for contouring the target as well as organs at risk. This can be overcome by megavoltage CT (MVCT) imaging.^(^ ^8^ ^)^ It has been observed that compared to kVCT, MVCT images exhibit considerably reduced artifacts in metal implants such as hip implants, surgical clips and dental fillings,^(^ ^9^ ^)^ and provide good agreement to measurements in dose prediction.^(^ ^10^ ^,^ ^11^ ^)^ Although MVCT scans are inferior in soft tissue contrast and spatial resolution compared to those of kVCT scans,^(^ ^12^ ^)^ they can be used for patient alignment and treatment planning as an alternative for the situations when kVCT scans are not available or are compromised by artifacts.^(^ ^13^ ^–^ ^17^ ^)^

StatRT is a new software package for the helical TomoTherapy Hi·Art (TomoTherapy Inc., Madison, WI) treatment planning system and is particularly important in time‐sensitive cases. The software is designed to manage scanning, planning and delivery in a timesaving manner. Conventionally, tomotherapy offers three options for plan optimization on the planning station: “TERMA”, “Full Scatter”, and “Beamlet”. The “TERMA” mode only accounts for the total energy released per unit mass (TERMA) of each beamlet during the process of optimization, without taking into account the transportation of charged particles and scattered particles. Thus, it is the fastest approach of the three, but not the most accurate. The Full Scatter mode calculates both TERMA as well as electron transportation in each iteration of optimization. It is the slowest but most accurate mode for dose optimization. The Beamlet mode allows users to “batch” beamlets for calculating dose contributions from each beamlet and optimize beamlet weights in each iteration with the real‐time display of the dose volume histograms. Thus, it is the most common option for conventional tomotherapy optimization. With StatRT, plans are still calculated using the same photon dose calculation engine, which is the convolution/superposition algorithm, and delivered in a helical IMRT manner to achieve highly conformal dose distributions. The differences from the conventional procedures are that the Full Scatter mode is the only option for plan optimization and all steps – including image acquisition, target and organ delineation, optimization, dose calculation and plan delivery – are performed on the operator station. It should eliminate the time for the physical transfer of patients from simulation to treatment and the data transfer from imaging to planning. An emergency palliation can be efficiently initiated with the StatRT approach on the operator station. Also, it offers operating ease as the entire process can be managed on a single computer, the Hi·Art treatment system's operator station. It is found that the dose distributions from StatRT are much more conformal than the traditional palliative techniques.^(^ ^18^ ^,^ ^19^ ^)^ This allows better tissue sparing and possibly better tolerance of the treatment.

However, there are several aspects yet to be studied, such as the shortcomings of this approach, the appropriate parameters for scanning and planning, and the timelines for the first fraction. The present planning study aimed to establish a clinical workflow and feasibility for performing the StatRT palliative treatment. The reported case is also a typical example where MVCT images are the superior option due to the presence of metal artifacts in kVCT. The treatment quality of StatRT is also compared with the conventional tomotherapy planning technique on a planning station (Tomo plan) in order to determine the advantages and disadvantages of both approaches.

## II. MATERIALS AND METHODS

### A. Patient history and diagnosis

A 72‐year‐old man was initially diagnosed and treated for stage one bronchoalveolar adenocarcinoma of left lung in 2006. Two years later he presented with back pains. CT images from a Philips Brilliance 40‐channel CT scanner (Philips Healthcare, Andover, MA) revealed a large lytic lesion at T10, consistent with metastatic disease. A bone scan showed radiotracer activity at T10. He underwent surgical intervention for what appeared to be impending spinal cord compression, specifically undergoing decompression of the spinal cord with laminectomy of T6 and T10 and a component of T11 with posterior instrumentation from T7 through L1 using stainless steel rods. The patient was prescribed for a course of palliative RT for the metastatic non‐small‐cell lung cancer, with the prescription regimen of 30 Gy in 10 fractions directed to T8 through T12.

### B. Image acquisition and treatment planning

**Figure 1:**
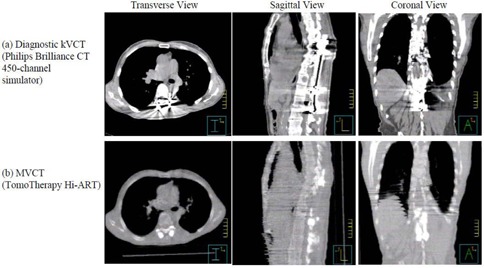
Two scans: diagnostic kVCT scan (a) and MVCT scan (b) on the TomoTherapy Hi·Art system for a patient with metal implants in the spine.

Significant metallic artifacts due to the stainless steel spine stabilizing rods were observed on the initial CT images (Fig. 1(a). These artifacts were determined too severe to allow treatment planning even with the density override. Alternatively, a MVCT image set (Fig. 1(b) was obtained using the 3.5 MV beam on the tomotherapy unit for treatment planning. Tomotherapy has two laser systems installed in the treatment room, with a fixed green laser system to indicate the virtual isocenter outside the bore and a movable red laser system to indicate the treatment position. When performing the image acquisition during the StatRT approach, the red lasers are in their “home” position (coinciding with the green) and projecting to the virtual isocenter. The virtual isocenter is defined outside the bore, 70 cm away from the actual isocenter in the longitudinal direction. Patients are usually aligned to the green lasers for anterior‐posterior and left‐right directions, but to the red laser on both sides for leveling. MVCT interslice distance can be selected as “coarse” (6 mm), “normal” (4 mm), and “fine” (2 mm).^(^ ^20^ ^)^ In this case, the MVCT scan took about nine minutes (around 520 seconds) using a “normal” grid and slice selection from −20 cm to +20 cm, with the central slice being the virtual setup isocenter, giving a total scan length of 40 cm. As we gained experience, we found that a shorter (around 7 minutes) scan, from −15 cm to +15 cm is often sufficient to cover most of the tumor sites. The artifacts could not be completely eliminated in the MVCT image but were substantially reduced in comparison to kVCT. The patient was sent home after MVCT acquisition and came back for a regular treatment using an approved and verified tomotherapy plan that was done in a conventional way. That is to say, the MVCT image set was exported to the Pinnacle planning system for contouring of the planning target volume (PTV), heart and lungs. It was then sent back to tomotherapy for conventional tomo planning using the Beamlet mode and 300 iterations(Tomo Plan 1). In our clinic, the image‐value‐to‐density table (IVDT) for MVCT scans was periodically verified and also acquired every time prior to a MVCT planning.

To test the feasibility of the StatRT planning on the operator station, the PTV, lungs and heart were also contoured on the MVCT images, with the auto‐contour tool used for lungs, and the StatRT plan was generated. It took approximately 15 minutes in contouring, mostly as a result of the complex PTV contoured by the physician. StatRT optimization with five iterations in the Full Scatter mode took around 10 minutes. For comparison, Tomo Plan 2 was also generated in the Full Scatter mode and five iterations to mimic the StatRT approach, but was completed on the planning station using the first set of contours as used for the Tomo Plan 1. Table 1 shows complete information on the parameters setup for StatRT and two Tomo plans. The field width and pitch remained the same for all three plans.

**Table 1:** Treatment planning parameter setup for StatRT and two Tomo plans.

	*StatRT Plan*	*Tomo Plan 1*	*Tomo Plan 2*
Optimization Mode	Full Scatter	Full Scatter	Beamlet
Iteration	5	5	300
Pitch	0.287	0.287	0.287
Field Width	5 cm	5 cm	5 cm
Modulatin Factor (actual)	2.7 (1.33)	2.7 (1.30)	2.7 (2.50)
Treatment Duration	203 sec	203 sec	376 sec
Gantry Period	15 sec	15 sec	27 sec

### C. Pretreatment verification and patient DQA following the first fraction

**Figure 2:**
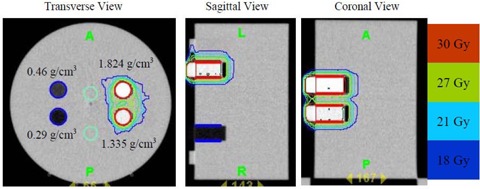
Transverse, sagittal and coronal views of the MVCT scan of the “cheese” phantom with two high‐density inserts ($1.335\;g/{\text{cm}}^{3}$ and $1.824\;g/{\text{cm}}^{3}$) and two low‐density inserts ($0.46\;g/{\text{cm}}^{3}$ and $0.29\;g/{\text{cm}}^{3}$). The two high‐density inserts are delineated in red as the PTV (prescribed to 30 Gy). The two low‐density inserts are delineated in blue as avoidance structures. Isdodose curves (100%, 90%, 70% and 60%) are also shown.

The tomotherapy “cheese” phantom with two high‐density and two low‐density plugs were MVCT scanned and planned using the StatRT software (Fig. 2). A hypothetical PTV was contoured on the high‐density plugs and given the same prescription dose as the patient treatment. Avoidance structures were contoured on the low‐density plugs. The delivery quality assurance (DQA) plan was generated on the same MVCT image and measured using the ion chamber/film system. This verification was created to test the dose delivery accuracy with density heterogeneity using MVCT scans prior to the patient treatment.

For a genuine clinical emergency with the patient scheduled to be treated immediately using the StatRT, the DQA would be conducted after the first fraction. DQA is fully integrated into the planning software, and it can be delivered from the operator station to compare with the planar chamber dose measurements using the OmniPro‐I'mRT MatriXX system (IBA Dosimetry, Schwarzenbruck, Germany) with the 2D ionization chamber array.^(^ ^21^ ^,^ ^22^ ^)^

## III. RESULTS

**Figure 3:**
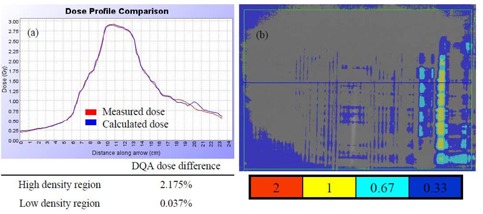
(a) Dose profile comparison between measured dose by ion chamber matrix and calculated dose; (b) gamma map for 3% / 3 mm criteria between the film measured dose and calculated dose.

**Figure 4:**
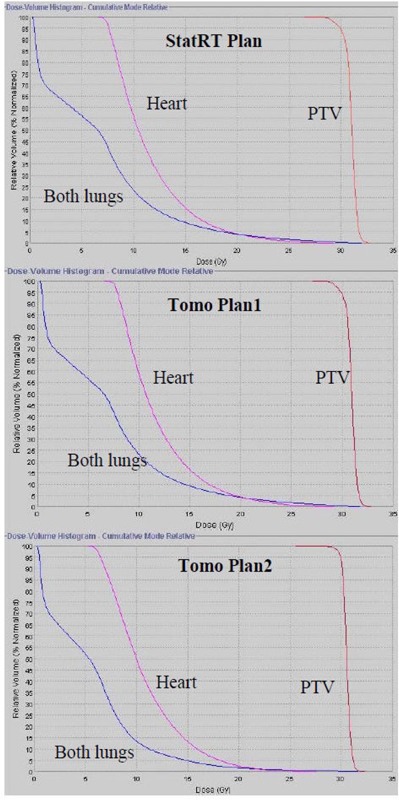
DVH comparisons for: (i) StatRT, (ii) Tomo Plan 1 and (iii) Tomo Plan 2. PTV, heart and lungs are illustrated by red, pink and blue, respectively.

**Figure 5:**
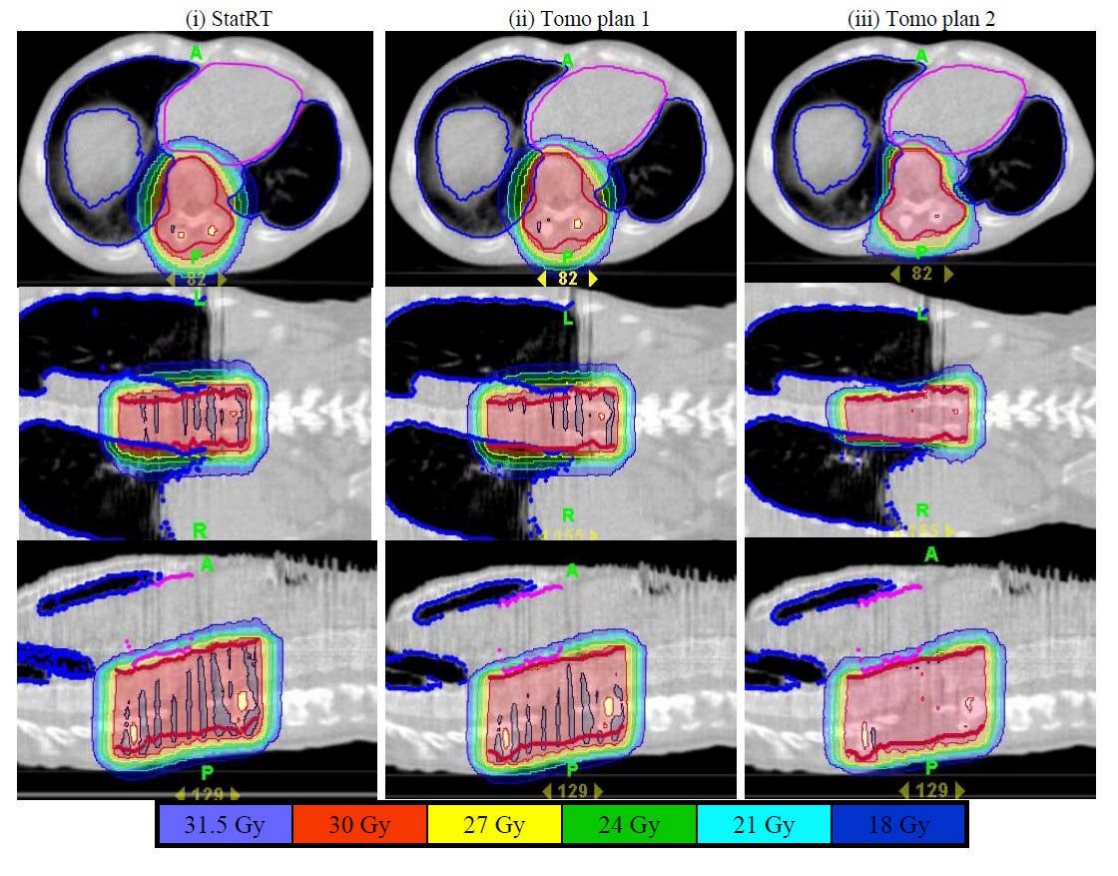
Comparison of six isodose levels (105%, 100%, 90%, 80%, 70% and 60%) for three plans with different plan settings: (i) StatRT plan, (ii) Tomo Plan 1 and (iii) Tomo Plan 2. Overall, all isodose lines are almost identical in the three plans, except for the 105% level where severe thread effects occurred in (i) and (ii).

**Figure 6:**
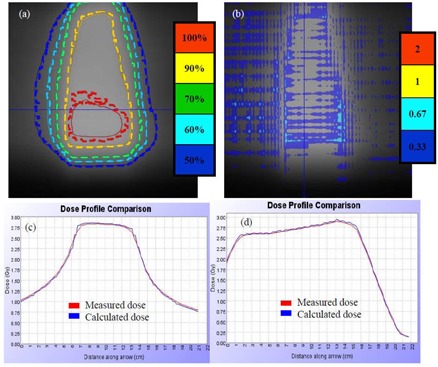
Following first fraction DQA for the patient treatment using the MatriXX system: (a) isodose comparison for levels 100%, 90%, 70%, 60%, and 50%; (b) gamma map with 3%/3 mm criteria for planer dose comparison; (c) and (d) lateral and longitudinal profile comparisons between measured (red) and calculated (blue) doses, respectively.

Figure 3(a) shows that the absolute dose difference is 2.175% at the high‐density region and 0.037% near the low‐density region. The gamma map on Fig. 3(b) showed a dose agreement within ±3%/3 mm between ion chamber/film measurements and dose calculation on the MVCT image set of the “cheese” phantom with density heterogeneity. The choice of the Beamlet mode and the high level of the intensity modulation resulted in longer gantry period and thus a longer treatment time (by 46%) for Tomo Plan 2 compared to the other two plans (Table 1). The dose parameters along with the homogeneity index for all three plans are calculated and tabulated in Table 2. The homogeneity index describes the target dose uniformity and is calculated by:

(1) $$\text{Homogeneity}\;\text{Indexes}=\frac{\left(D_{2}-D_{98}\right)}{D_{p}}\times 100\%$$

where $D_{2}$ and $D_{98}$ represent the doses to 2% and 98% of the PTV, and $D_{p}$ represents the prescription dose^(^ ^23^ ^)^ Dose analysis in Table 2 reveals that these three planning methods yield comparable results for max/min/mean doses to heart, lungs, and PTVs. The max/min/mean doses to PTV in the StatRT plan are comparable to Tomo plan 1 and 2. However, the homogeneity index comparison shows a significant improvement in dose homogeneity in Tomo Plan 2 compared to StatRT and Tomo Plan 1, due to the reduced thread effects with higher number of iterations. Doses to the heart and lungs are within clinically acceptable dose limits in the StatRT and the two Tomo plans. Dose volume histograms (DVHs) of the three plans are shown in Fig. 4. The DVH of the StatRT plan is almost identical to Tomo Plan 2. Highly conformal dose coverage to the tumor site was achieved in all three plans. Six isodose levels – 105%, 100%, 90%, 80%, 70% and 60% – were compared (Fig. 5). The isodose lines are identical except for the 31.5 Gy dose level (i.e., 105% of the prescription), represented as a distinct “thread effect”, which resulted from the undermodulation in the StatRT plan and Tomo Plan 1 where only five iterations were run in optimization. The thread effects in this case present as a series of dose ripples near the outer edge of dose distribution along the longitudinal direction. The results show that the radiation dose distributions in the StatRT planning are equivalent to a Tomo plan with the same optimization parameters. Also, we observed that treatment time is significantly shorter when less iteration is used in the planning process. The overall time for the treatment process (including 7 minutes for simulation, 15 minutes for contouring, 10 minutes for planning and 5 minutes for delivery) decreases from hours to around 40 minutes using the StatRT procedure. DQA performed for the StatRT plan is shown in Fig. 6. The calculated dose from the StatRT plan was verified to be within 3%/3 mm agreement with the measured dose.

**Table 2:** Dose index comparisons for: (i) the StatRT plan, (ii) the Tomo Plan 1 and (iii) the Tomo Plan 2.

		*Max Dose (Gy)*	*Min Dose (Gy)*	$D_{40\%}$ *(Gy)*	*Mean Dose (Gy)*	*Homogeneity Index*	*Physical Volume (cc)*
(i)	PTV	32.9	26.7	‐	31.1	9.0	504.6
	Heart	29.3	6.7	10.2	11.6	‐	436.3
	Lungs	31.9	0.3	5.6	6.8	‐	3892.0
(ii)	PTV	32.6	27.0	‐	31.0	6.0	509.8
	Heart	29.2	6.7	10.2	11.8	‐	428.9
	Lungs	31.7	0.3	5.6	6.8	‐	3925.5
(iii)	PTV	32.2	25.6	‐	30.6	2.1	509.8
	Heart	27.6	5.4	10.1	10.9	‐	428.9
	Lungs	31.7	0.3	5.3	5.6	‐	3925.5

## IV. DISCUSSION

Palliative radiotherapy is generally appropriate when there are slim chances of cure and the clinical aim is for pain control or other symptomatic relief. During palliative radiotherapy it becomes important to efficiently synchronize the scanning, treatment planning, contouring, calculations or simulation‐associated statistical data, and treatment delivery in an efficient manner. StatRT is an extended utility on tomotherapy for urgent situations when time is a significant constraint. This efficiency is invaluable for facilities that have only one tomotherapy unit or circumstances in which urgent palliation is considered desirable. With the current toolset, it is possible to scan, plan and deliver radiotherapy treatment within one hour in comparison to the ordinary situation that takes hours of waiting time between simulation and treatment. A shorter planning window used in the StatRT plan still allows at least five iterations of optimization, which generates plans with clinically acceptable quality. Yet thread effects are distinct without sufficient iterations of optimization. Using recommended pitch factors (0.86/n) helps in minimizing the them.^(^ ^24^ ^)^ Nevertheless plans with a $D_{1\%}$ value smaller than 110% and homogeneity index smaller than 17 are still classified “good” or “outstanding”, based on the RTOG criteria.^(^ ^25^ ^)^ Further study is needed to understand the clinical significance of the heterogeneous dose distribution with the thread effects. Though higher number of iterations might improve plan quality, it is at the cost of increased planning and delivery time. The simulation time can be restricted to 10 minutes using the “normal” mode and the contouring time can be reduced also as more experience accumulated. Total time from scanning to treatment can be reduced to 40 minutes, facilitated by the use of a fast auto‐contouring tool and fewer (<10) iterations in optimization. Comparable DVHs and dose distributions were observed in the Tomo plan using the “Full Scatter” optimization setting. The StatRT technique is beneficial to the patients in need of palliative treatment by reducing the pain from being moved around.

Typically, AP‐PA sets of beams are used for such a palliative treatment since the intended doses can be achieved with a homogenous dose distribution.^(^ ^26^ ^)^ Generating a palliative plan takes much less time on a conventional linear accelerator than the procedure described above. The portal imager or a conventional kilovoltage X‐ray simulator is normally used where the radiation oncologist specifies field borders and designs beam blocks to isolate the tumor from critical sensitive organs. However, in our facility only one tomotherapy machine is available and the volumetric CT image is required for treatment planning. This increases difficulties in palliative treatment especially for a case as described in this study. It is noted that the absorbed dose in the soft tissue regions affected by the high‐density artifacts is higher in the kVCT image than the MVCT‐based recalculation. The dose differential can be as large as 15%.^(^ ^27^ ^)^ Due to the mild appearance of the metallic streak artifacts, MVCT provides better image quality in the region surrounding the metal hardware. The MVCT image reveals sufficient information of the tumor and normal anatomy for patient repositioning and treatment planning.^(^ ^10^ ^,^ ^11^ ^,^ ^28^ ^)^ It is worth noting that recent studies show output instabilities over time of the tomotherapy imaging beam and the consequent dose effect can be as large as 3%.^(^ ^29^ ^,^ ^30^ ^)^ Therefore, our protocol requires acquisition of a new IVDT prior to every MVCT scans for dose calculation purpose.

It might be difficult for patients with significant pain to remain in the same position for more than 30 minutes; thus we recommend a second MVCT scan prior to the treatment in order to verify the target position. The time for this additional MVCT scan was not included in the total time mentioned above. We would expect another 5–10 minutes, depending on the length of the target, the choice of the scan mode and the difficulty in registration. The entire procedure takes 40 minutes to one hour, which limits the usage of the tomotherapy unit for other patients. It is also important to coordinate the schedule with all personnel involved, including physician and medical physicist.

## V. CONCLUSIONS

In situations where conventional kVCT is unable to provide accurate anatomical information due to interference from high‐density materials such as metal, MVCT‐based planning can be advantageous. With the successful integration of MVCT on the workstation, tomotherapy offers two options for treatment planning using either Tomo plan or StatRT plan. Satisfying essentially all of the treatment planning objectives, the StatRT technique has proved to be an excellent solution for combining image acquisition, treatment planning and dose delivery in one session when time is the limiting factor. The dose distribution from StatRT appears equivalent to a Tomo plan with a limited number of iterations. It also appears comparable to a Tomo plan with large number of iteration except for the presence of a noticeable “thread effect” at high isodose levels. In conjunction with MVCT imaging, StatRT is a feasible tool for physicians to scan, contour and treat patients within one hour, and can be beneficial when there are severe imaging artifacts in the target area.
